# Hypoxic conditioning of neuroblastoma cells promotes CD8^+^ T-cell dysfunction through the HIF-1*α*/PD-L1 axis

**DOI:** 10.3389/fped.2026.1923893

**Published:** 2026-10-14

**Authors:** Yujin Chao, Guili Zheng

**Affiliations:** 1 Department of Pediatrics, the 960th Hospital of PLA Joint Logistics Support Force, Jinan, Shandong, China; 2 Department of Oncology, the 960th Hospital of PLA Joint Logistics Support Force, Jinan, Shandong, China

**Keywords:** exhaustion-like phenotype, HIF-1*α*, hypoxia, neuroblastoma, PD-l1, T cell dysfunction

## Abstract

**Objective:**

To investigate whether hypoxic conditioning of neuroblastoma (NB) cells induces CD8^+^ T cell dysfunction/exhaustion-like phenotype via the HIF-1*α*/PD-L1 axis and whether targeting this axis reverses immune suppression.

**Methods:**

Human NB cell lines SK-N-SH, SK-N-BE(2), and SK-N-AS were exposed to 1% O₂ hypoxia. HIF-1*α* and PD-L1 expression were assessed by Western blot, qRT-PCR, and flow cytometry. ChIP-qPCR and dual-luciferase assays were used to verify HIF-1*α* binding and transcriptional activation of the PD-L1 promoter. SK-N-SH cells pre-conditioned under normoxia or hypoxia were co-cultured with primary human CD8^+^ T cells. CD8^+^ T cell dysfunction/exhaustion-like phenotype (PD-1^+^TIM-3^+^), apoptosis, IFN-*γ*, Granzyme B, CD107a, and specific cytotoxicity were evaluated. HIF-1*α* siRNA, YC-1, and PD-L1 neutralizing antibody were applied. The direct effect of YC-1 on T cells was also assessed.

**Results:**

Hypoxia increased HIF-1*α* and PD-L1 expression in a time-dependent manner and also upregulated PD-L1 in additional NB cell lines. ChIP-qPCR and dual-luciferase assays confirmed direct HIF-1*α* binding to the HRE (5’-AGGTGC-3’) and transcriptional activation. PD-L1 elevation declined after reoxygenation. Hypoxia-preconditioned NB cells increased PD-1^+^TIM-3^+^ CD8^+^ T cells (5.49%–32.24%) and apoptosis (10.61%–26.89%), while reducing IFN-*γ*^+^, Granzyme B^+^, CD107a^+^ cells and specific cytotoxicity (79.94%–38.40%). HIF-1*α* knockdown or PD-L1 blockade alleviated these effects. YC-1 at 25 μM did not significantly affect T cell viability or IFN-*γ* secretion.

**Conclusion:**

Hypoxic conditioning of NB cells stabilizes HIF-1*α*, enhances its binding to the PD-L1 promoter HRE, upregulates PD-L1, and induces CD8^+^ T cell dysfunction/exhaustion-like phenotype and impaired antitumor function. Targeting HIF-1*α* or PD-L1 partially restores T cell function in this co-culture model. The hypoxia-driven HIF-1*α*/PD-L1 axis contributes to immune suppression in NB and may be a potential therapeutic target.

## 1 Introduction

Neuroblastoma (NB) is the most common extracranial solid tumor in children. It comes from nerve crest cells during sympathetic nervous system development and often grows in the adrenal gland or sympathetic ganglia. NB causes nearly 15% of childhood cancer deaths (1–3). Even with intensive treatments like surgery, chemotherapy, bone marrow ablation with stem cell support, and radiotherapy, the outcome for high-risk NB patients remains poor (4, 5).

Immune checkpoint inhibitors (such as anti-PD-1/PD-L1 antibodies) have worked very well in adult cancers like melanoma and non-small cell lung cancer (6, 7). But their clinical response rate in NB is much lower than expected. High PD-L1 levels are linked to high-risk NB and poor prognosis (8), but the clinical benefit of anti-PD-L1 monotherapy in NB is still unclear (9). Preclinical studies in NB mouse models and early clinical trials of anti-PD-1/anti-PD-L1 alone did not show survival benefits (10–12). However, two patients with refractory NB did respond to a combination of anti-PD-1 and anti-GD-2 therapy (13). These findings suggest that NB may have unique immune escape mechanisms that cause primary resistance to immune checkpoint inhibitors.

The hypoxic tumor microenvironment is a key driver of immune escape and therapy resistance (14, 15). Fast tumor growth and abnormal blood vessel development often create obvious hypoxic areas inside NB tissues. Under normal oxygen conditions, HIF-1*α* is hydroxylated by prolyl hydroxylases and subsequently directed to proteasomal degradation. In contrast, hypoxia stabilizes HIF-1*α*, enabling its nuclear translocation and transcriptional activation of diverse downstream genes (16). PD-L1 is an important target of HIF-1*α*. Noman and colleagues first showed that the PD-L1 gene promoter contains a functional HRE, and HIF-1*α* can directly bind to this element to induce PD-L1 transcription (17). In many solid tumors, hypoxia upregulates PD-L1 via HIF-1*α*, which then binds to PD-1 on T cells, suppressing T cell activation, proliferation, and effector functions, and pushing T cells toward a dysfunctional or exhaustion-like state (18). T cell exhaustion is marked by elevated co-inhibitory receptors (e.g., PD-1 and TIM-3), reduced production of effector cytokines (IFN-*γ*, Granzyme B), and loss of killing ability (19). However, in NB, whether hypoxic conditioning of NB cells induces CD8^+^ T cell dysfunction through the HIF-1*α*/PD-L1 axis, and whether targeting this axis can reverse immune suppression, still lacks systematic functional evidence. One study has shown that hypoxia induces HIF-1*α*-dependent PD-L1 upregulation in NB cell lines (20), but the exact impact of this signaling axis on CD8^+^ T cell function and its potential as a therapeutic target remain to be fully explored.

Based on this background, we established an *in vitro* hypoxia model using human NB cell lines. By combining gene silencing, pharmacological inhibition, chromatin immunoprecipitation, dual-luciferase reporter assays, and co-culture of tumor cells with primary human CD8^+^ T cells, we investigated whether hypoxia stabilizes HIF-1*α* and upregulates PD-L1, whether HIF-1*α* directly binds the HRE in the PD-L1 promoter and activates its transcription, and whether hypoxia-pretreated NB cells induce a CD8^+^ T-cell dysfunction/exhaustion-like phenotype and impaired antitumor function through the HIF-1*α*/PD-L1 axis. We further examined whether pharmacological inhibition of HIF-1*α* or PD-L1 blockade reverses these suppressive effects. This study aimed to investigate whether hypoxic conditioning of NB cells induces a CD8^+^ T-cell dysfunction/exhaustion-like phenotype through the HIF-1*α*/PD-L1 axis, and to provide preliminary *in vitro* evidence for future studies targeting this axis.

## 2 Materials and methods

### 2.1 Cell culture and hypoxia treatment

The human NB cell line SK-N-SH, SK-N-BE(2) (MYCN-amplified), and SK-N-AS (MYCN-non-amplified) were obtained from ATCC. Cells were maintained in high-glucose DMEM supplemented with 10% FBS (Gibco) and 1% penicillin/streptomycin (Gibco) at 37 °C in a 5% CO_2_ humidified incubator. Only log-phase cells were used.

To induce hypoxia, cells were seeded in 6-well plates at 2 × 10^5^ per well. After overnight attachment, they were transferred to a tri-gas incubator (1% O_2_, 5% CO_2_, 94% N_2_) for 6, 12, 24, or 48 h. Normoxic controls were kept in 21% O_2_, 5% CO_2_ and collected at the same time points. For validation in additional NB cell lines, SK-N-BE(2) and SK-N-AS were cultured under the same conditions and subjected to normoxia or hypoxia (1% O₂) for 24 h before qPCR and Western blot analysis.

To assess the effect of culture time and confluence on PD-L1 expression under normoxia, SK-N-SH cells were cultured under normoxia (21% O₂, 5% CO₂) for 0, 6, 12, 24, and 48 h. For reoxygenation experiments, SK-N-SH cells were exposed to hypoxia (1% O₂, 5% CO₂, 94% N₂) for 24 h and then returned to normoxia for 0, 6, 12, and 24 h. Total RNA and protein were extracted at the indicated time points and subjected to qPCR and Western blot analysis.

### 2.2 Western blot

Cells were lysed in ice-cold RIPA buffer containing protease and phosphatase inhibitor cocktails for 30 min, followed by centrifugation at 4 °C. Protein concentration was measured by BCA assay. 30 μg of protein were resolved by 10% SDS-PAGE and electrotransferred onto PVDF membranes. After blocking with 5% non-fat milk for 1 h, membranes were probed overnight at 4 °C with primary antibodies: anti-HIF-1*α* (1:1000), anti-PD-L1 (1:1000), or anti-*β*-actin (1:1000). The next day, HRP-conjugated secondary antibodies (1:2000) were added for 1 h at room temperature. Bands were visualized by ECL and quantified using ImageJ; *β*-actin was used as the loading control.

### 2.3 Real-time quantitative PCR (RT-qPCR)

Total RNA was extracted with TRIzol, and its concentration/purity was measured by NanoDrop. 1 μg of RNA was reverse-transcribed into cDNA using PrimeScript RT Master Mix (TaKaRa). qPCR was conducted with SYBR Green Master Mix under the following cycling parameters: 95 °C for 10 min, followed by 40 cycles of 95 °C for 15 s and 60 °C for 60 s. *β*-actin was employed as the internal control, and relative expression was computed via the 2⁻*ΔΔ*Ct method. The primer sequences are listed in Table 1.

**Table 1:** Primer sequences (5’−3’).

Gene	Forward primer	Reverse primer
PD-L1	GGACAAGCAGTGACCATCAAG	CCCAGAATTACCAAGTGAGTCCT
CA9	GGATCTACCTACTGTTGAGGCT	CATAGCGCCAATGACTCTGGT
VEGF	TTGCCTTGCTGCTCTACCTCCA	GATGGCAGTAGCTGCGCTGATA
*β*-actin	GGTGGCTTTTAGGATGGCAAG	ACTGGAACGGTGAAGGTGACAG

### 2.4 Flow cytometry for surface PD-L1

After hypoxia or normoxia treatment, cells were trypsinized, washed in cold PBS, and resuspended in PBS containing 2% FBS at 1 × 10^6^/mL. They were stained with PE-anti-human PD-L1 (1:50, Elabscience, #E-AB-F1133D) for 30 min at 4 °C in the dark. After double washing, cells were run on a flow cytometer, and FlowJo was employed to calculate the PD-L1-positive rate; isotype control was used to set the gate.

### 2.5 Cell viability assay

Cells were incubated with CCK-8 reagent for 2 h at 37 °C, and absorbance at 450 nm was measured.

### 2.6 HIF-1*α* siRNA transfection

Two independent siRNAs targeting HIF-1*α* (siHIF-1*α*#1 sense: 5’-UGATACCAACAGUAACCA A(dTdT)-3’; anti-sense: 5’-UUGGUUACUGUUGGUAUCA(dTdT)-3’ or siHIF-1*α*#2 sense: 5’-CAUGAAAGCACAGAUGAAU(dTdT)-3’; anti-sense: 5’-AUUCAUCUGUGCUUUCAUG(dTdT)-3’) and siControl were synthesized by Shanghai GenePharma. Cells were seeded in 6-well plates (2 × 10^5^/well) to reach 50%–60% confluency on the day of transfection. Two hours prior to transfection, the medium was replaced with serum-free Opti-MEM. Lipofectamine RNAiMAX and siRNA were each diluted in Opti-MEM, mixed, and incubated for 5 min at room temperature to form complexes. The mixture was added dropwise to each well (final siRNA concentration 50 nM). After 6 h, the medium was replaced with complete growth medium. At 48 h post-transfection, cells were exposed to normoxia or hypoxia. Knockdown efficiency (≥70% reduction in HIF-1*α* protein) was verified by Western blot.

### 2.7 YC-1 pharmacological inhibition

The HIF-1*α* inhibitor YC-1 (Selleck, #S7958) was dissolved in DMSO as a 50 mM stock. SK-N-SH cells were treated with YC-1 (10, 25, 50 μM) or equal volume of DMSO for 48 h under normoxia or 1% O₂. Cells were then collected for Western blot and flow cytometry.

### 2.8 Chromatin immunoprecipitation-qPCR (ChIP-qPCR)

To probe HIF-1*α* binding to the PD-L1 promoter, we conducted ChIP-qPCR. Hypoxia-treated (24 h) SK-N-SH cells were crosslinked (1% formaldehyde, 10 min) and quenched with glycine. Lysates were digested with micrococcal nuclease and sonicated to generate 200–1,000 bp chromatin fragments. Equal amounts of sheared chromatin were incubated overnight at 4 °C with anti-HIF-1*α* or control IgG, then pulled down with protein G magnetic beads for 2 h. After rigorous washing, bound DNA was eluted, decrosslinked, and purified. qPCR assessed enrichment at the PD-L1 HRE and a distal reference region; results are presented as fold change over the IgG control.

### 2.9 Dual-luciferase reporter assay

Firefly luciferase reporter plasmids driven by wild-type (5’-AGGTGC-3’) or HRE-mutant (5’-AAAAAC-3’) PD-L1 promoter sequences were commercially synthesized by GeneChem (Shanghai, China). SK-N-SH cells were co-transfected with one reporter construct and pRL-TK (Renilla luciferase internal control). Twenty-four hours after transfection, cells were incubated under normoxia or 1% O₂ hypoxia for 24 h. Cells were then lysed, and luciferase activities were measured using the dual-luciferase reporter assay system. Firefly luminescence was normalized to Renilla luminescence; relative activity was calibrated by setting the WT normoxia group to 1.0. Three independent biological experiments were performed with three technical replicates per experiment. Statistical analysis was carried out using two-way ANOVA followed by Tukey's post-hoc test.

### 2.10 Co-culture of tumor cells with CD8^+^ T cells and T cell functional assays

#### 2.10.1 CD8^+^ T cell thawing and activation

Primary human CD8^+^ T cells were commercially obtained from IPHASE (Shanghai, China) and isolated by magnetic negative selection (purity ≥85%). According to the manufacturer, cells were obtained from healthy donors who provided written informed consent, and donors were screened negative for HBV, HCV, HIV, and syphilis. Cells were recovered from cryopreservation in RPMI 1,640 supplemented with 10% FBS. After centrifugation, cells were resuspended in the same medium containing 100 U/mL IL-2 at 1 × 10^6^/mL (viability ≥85%). Prior to co-culture, cells were stimulated for 72 h with plate-immobilized anti-CD3 (1 μg/mL), soluble anti-CD28 (1 μg/mL), and IL-2 (100 U/mL). These T cells were polyclonally activated and were not tumor-antigen-specific. The exact number of individual donors is not disclosed by the vendor; therefore, inter-donor variability could not be assessed.

#### 2.10.2 Tumor cell pretreatment and co-culture

SK-N-SH cells were pretreated for 48 h under: (1) Normoxia; (2) Hypoxia (1% O_2_); (3) Hypoxia + siNC; (4) Hypoxia + siHIF-1*α*; (5) Hypoxia + isotype (10 μg/mL); (6) Hypoxia + *α*-PD-L1 (10 μg/mL). Rescue groups: (7) Hypoxia + YC-1 (25 μM); (8) Hypoxia + *α*-PD-L1. YC-1 was added during the 48 h pretreatment and removed by washing before co-culture. *α*-PD-L1 or isotype was added at the beginning of co-culture and maintained throughout. After pretreatment, tumor cells were washed, labeled with CFSE (5 μM), and co-cultured with activated CD8^+^ T cells at a 5:1 ratio under normoxia for 48 h.

In this model, tumor cells were pre-conditioned under hypoxia and then co-cultured with T cells under normoxia.

#### 2.10.3 Analysis of CD8^+^ T cell dysfunction/exhaustion-like phenotype

After co-culture, T cells were stained with Live/Dead dye to exclude dead cells, washed, and then stained with a surface antibody mix: PerCP-anti-CD3 (1:50), PE-anti-CD8 (1:50, Elabscience, #E-AB-F1104D), FITC-anti-PD-1 (1:50, Elabscience, #E-AB-F1229C), and anti-TIM-3 (1:50, HuaAn, #HA723169) for 30 min at 4 °C. After washing, rabbit secondary antibody (1:200, Servicebio, #GB21303) was added for another 30 min. Samples were analyzed on a flow cytometer. Gating strategy: lymphocytes → single cells → live cells → CD3^+^CD8^+^ T cells. Within this population, the percentage of PD-1^+^TIM-3^+^ cells and MFI of each marker were recorded.

#### 2.10.4 T cell apoptosis assay

To evaluate apoptosis, co-cultured CD8^+^ T cells were stained for CD3/CD8 and then incubated with Annexin V-FITC/PI (Lianke Bio, #AP101) in 100 μL 1 × Binding Buffer (5 μL Annexin V + 10 μL PI, 5 min, room temperature, dark). After adding 400 μL buffer, samples were analyzed within 1 h. Apoptosis was expressed as the % of Annexin V^+^ cells among CD3^+^CD8^+^ lymphocytes. Apoptosis was measured separately from PD-1/TIM-3 staining.

#### 2.10.5 T cell killing assay

For the killing assay (CFSE/PI kit, BioLab #HR8302), CFSE-labeled target cells were mixed with T cells (5:1, 6 h), then stained with PI (1 μg/mL, 5 min, dark) and subjected to flow cytometry. The percentage of CFSE^+^PI^+^ cells among total CFSE^+^ target cells was calculated to determine T-cell-mediated target cell killing. Spontaneous target cell death was determined by incubating CFSE-labeled target cells alone under the same conditions. Specific cytotoxicity was calculated as: Specific cytotoxicity (%) = [(experimental CFSE^+^PI^+^ %−spontaneous CFSE^+^PI^+^ %)/(100−spontaneous CFSE^+^PI^+^ %)] × 100.

#### 2.10.6 Effector function and degranulation assay

During the last 4–6 h of co-culture, PMA (50 ng/mL), ionomycin (1 μg/mL), Brefeldin A (1:1000), and PE-Cy5-anti-CD107a (Elabscience, #E-AB-F1149C) were added. After co-culture, T cells were fixed, permeabilized, and stained intracellularly with FITC-anti-IFN-*γ* (Elabscience, #E-AB-F1196D) and PE-anti-Granzyme B (Elabscience, #AN00961D). Flow cytometry was used to analyze the percentages of cytokine-positive and CD107a-positive cells within the CD3^+^CD8^+^ population.

### 2.11 Direct effect of YC-1 on CD8^+^ T cells

Primary human CD8^+^ T cells were treated with YC-1 (0, 10, 25, 50 μM) for 48 h. Cell viability was measured by CCK-8 assay. For IFN-*γ* detection, cells were stimulated with PMA (50 ng/mL) and ionomycin (1 μg/mL) for 4–6 h, and culture supernatants were collected for ELISA. Three independent experiments were performed, each with three technical replicates. Data are normalized to the 0 μM control (set as 100%) and presented as mean ± SD. Statistical analysis was performed using one-way ANOVA followed by Dunnett's *post hoc* test.

### 2.12 Statistical analysis

Data are shown as mean ± SD. All experiments were performed with at least three independent biological replicates unless otherwise indicated. For cell-based assays, independent biological replicates were performed on separate days. Two-group comparisons were made using two-tailed unpaired Student's t-test. Multiple groups were compared by one-way ANOVA followed by Tukey's test, and two-way ANOVA with Sidak's test was used for factorial designs. *p* < 0.05 was considered significant. All analyses were performed with GraphPad Prism 10.1.2.

## 3 Results

### 3.1 Hypoxia induces HIF-1*α* protein stabilization and upregulation of PD-L1 expression in SK-N-SH cells

To investigate the effects of hypoxia on HIF-1*α* stability and PD-L1 expression in NB cells, SK-N-SH cells were cultured under 1% O₂ conditions for varying durations (6, 12, 24, and 48 h). Western blot results showed that HIF-1*α* protein was virtually undetectable under normoxic conditions. Following hypoxic treatment, its levels rose rapidly, increasing to 1.63 ± 0.31 times that of the normoxic group after 6 h, 4.90 ± 0.48 times after 12 h, and reaching a peak of 6.56 ± 0.56 times after 24 h. Although levels decreased slightly after 48 h, they remained at 4.66 ± 0.43 times. PD-L1 protein expression showed a progressive upregulation, with a statistically significant increase beginning at 12 h (4.04 ± 0.21-fold) and reaching 9.44 ± 0.35-fold at 48 h (Figure 1A). qRT-PCR revealed that the relative expression levels of PD-L1 mRNA increased to 3.42 ± 0.38-fold and 5.16 ± 0.52-fold of the normoxic group after 24 and 48 h of hypoxia, respectively (Figure 1B).

**Figure 1:**
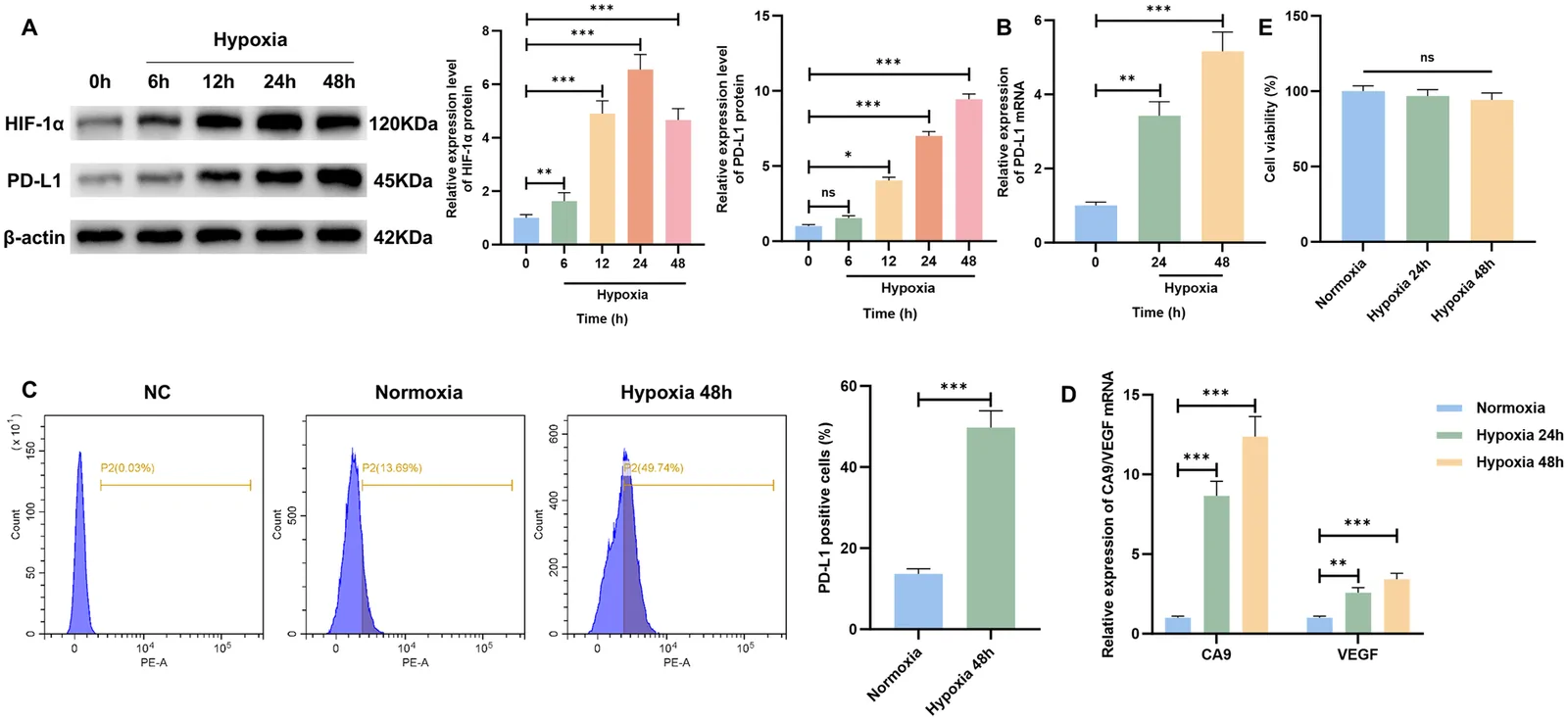
Hypoxia induces HIF-1*α* protein stabilization and upregulation of PD-L1 expression in SK-N-SH cells. **(A)**. Western blot analysis of HIF-1*α* and PD-L1 protein expression levels after treatment with normoxia or hypoxia for different durations (6, 12, 24, and 48 h). **(B)**. qRT-PCR analysis of relative PD-L1 mRNA expression levels following normoxic or hypoxic treatment. **(C)**. Flow cytometry analysis of surface PD-L1 expression levels 48 h after normoxic or hypoxic treatment. **(D)**. qRT-PCR analysis of hypoxia-induced changes in mRNA expression of the classical HIF-1*α* downstream target genes CA9 and VEGF. **(E)**. Cell viability was assessed by CCK-8 assay 24 and 48 h after treatment with normoxia or hypoxia. *p < 0.05, **p < 0.01, ***p < 0.001.

PD-L1 expression on the cell surface revealed that the percentage of positive cells was 13.69% ± 1.25% under normoxic conditions, which significantly increased to 49.74% ± 4.12% after 48 h of hypoxia (Figure 1C).

The mRNA levels of CA9 and VEGF, classic HIF-1*α* downstream target genes, also increased significantly after hypoxia. After 24 h, they rose to 8.65 ± 0.92-fold and 2.58 ± 0.31-fold, respectively, compared to the normoxic group, and further increased to 12.38 ± 1.25-fold and 3.42 ± 0.38-fold after 48 h (Figure 1D).

CCK-8 assays showed that relative cell viability at 24 and 48 h of hypoxia was 96.85% ± 4.21% and 94.18% ± 4.68% of that in the normoxic group, respectively, with no statistically significant difference, ruling out interference from hypoxic toxicity (Figure 1E).

To validate the generality of hypoxia-induced PD-L1 upregulation, we examined two additional NB cell lines: SK-N-BE(2) (MYCN-amplified) and SK-N-AS (MYCN-non-amplified). Hypoxia increased PD-L1 mRNA by approximately 2.7-fold in SK-N-BE(2) and 2.0-fold in SK-N-AS (Supplementary Figure S1A), and PD-L1 protein by approximately 2.4-fold and 1.7-fold, respectively, with concomitant HIF-1*α* accumulation (Supplementary Figure S1B). Thus, hypoxia-induced PD-L1 upregulation is not restricted to SK-N-SH cells.

To exclude effects of culture time and confluence, SK-N-SH cells were cultured under normoxia for 0–48 h; PD-L1 protein and mRNA remained unchanged (Supplementary Figures S2A,B). After 24 h of hypoxia followed by reoxygenation, PD-L1 levels gradually declined and approached baseline by 24 h (Supplementary Figures S2C,D), indicating that hypoxia-induced PD-L1 upregulation is reversible.

### 3.2 HIF-1*α* is an essential regulator of hypoxia-induced upregulation of PD-L1

Two strategies-siRNA and pharmacological inhibition-were employed to validate the regulatory role of HIF-1*α* on PD-L1. Following transfection with two independent siHIF-1*α* sequences, hypoxia-induced HIF-1*α* protein accumulation was significantly suppressed, with knockdown efficiencies of 72.18% and 74.06%, respectively (Figure 2A). qRT-PCR analysis revealed that in the hypoxia + siControl group, PD-L1 mRNA levels increased to 4.92 ± 0.48 times those of the normoxia group, whereas in the siHIF-1*α*#1 and siHIF-1*α*#2 groups, they decreased to 1.62 ± 0.22 and 1.45 ± 0.19, respectively (Figure 2B). Western blot analysis further confirmed that following HIF-1*α* knockdown, total PD-L1 protein levels decreased from 4.64 ± 0.35 to 1.61 ± 0.18 and 1.26 ± 0.16, respectively (Figure 2C). Flow cytometry results showed that the percentage of cells positive for surface PD-L1 decreased from 36.98% ± 3.85% to 12.70% ± 1.72% and 11.08% ± 1.55%, respectively (Figures 2D,E). The results from the two independent siRNAs were highly consistent, ruling out off-target effects.

**Figure 2:**
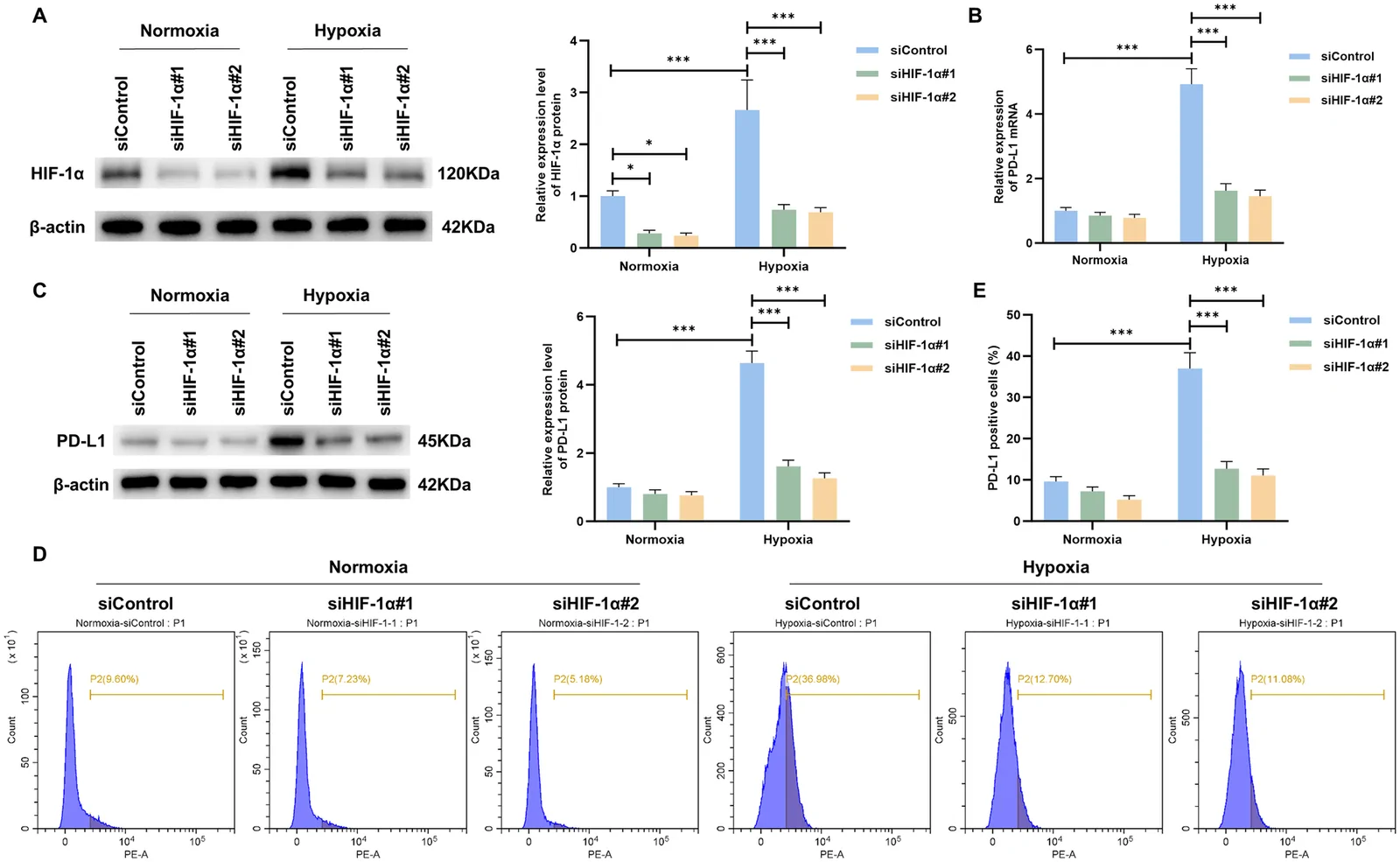
HIF-1*α* is a necessary regulatory factor for hypoxia-induced upregulation of PD-L1 (siRNA knockdown). **(A)**. Western blot analysis of HIF-1*α* protein expression levels under normoxic or hypoxic conditions following transfection with siControl or two independent siHIF-1*α* constructs. **(B)**. qRT-PCR analysis of relative PD-L1 mRNA expression levels in each group. **(C)**. Western blot analysis of total PD-L1 protein expression levels in each group. **(D**-**E)**. Flow cytometry analysis of the percentage of cells expressing surface PD-L1 in each group. *p < 0.05, ^***^p < 0.001.

SK-N-SH cells cultured under hypoxic conditions were treated with the HIF-1*α*-selective inhibitor YC-1. Western blot demonstrated that YC-1 treatment reduced HIF-1*α* protein levels in a dose-dependent fashion. Specifically, after exposure to 10, 25, and 50 μM of the inhibitor, the relative HIF-1*α* expression dropped to 3.07 ± 0.40, 2.47 ± 0.25, and 1.20 ± 0.16, respectively. Concurrent with the decrease in HIF-1*α*, PD-L1 protein expression decreased synchronously, dropping to 3.41 ± 0.26, 1.88 ± 0.20, and 1.33 ± 0.14 in the 10, 25, and 50 μM groups, respectively (Figure 3A).

**Figure 3:**
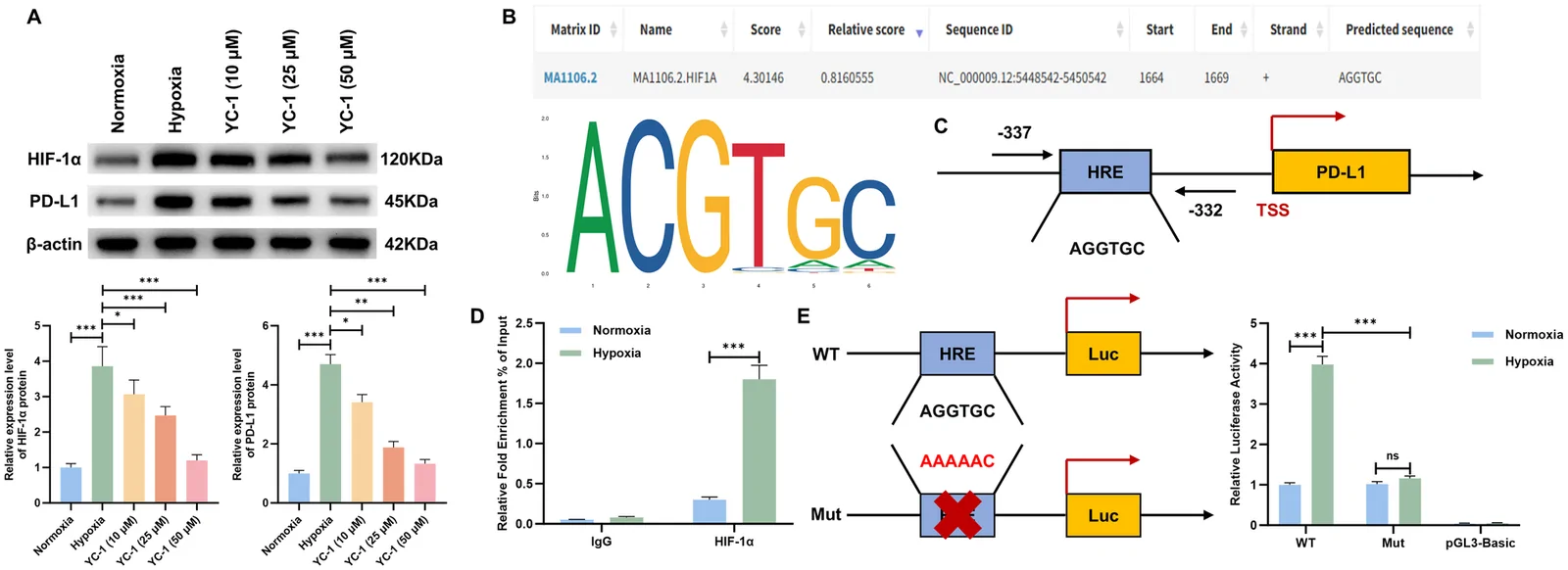
Pharmacological inhibition of HIF-1*α* by YC-1 reduces PD-L1 expression, and HIF-1*α* directly binds to the HRE in the PD-L1 promoter. **(A)**. Western blot analysis of HIF-1*α* and PD-L1 protein expression levels under hypoxic conditions following treatment with different concentrations of YC-1 (10, 25, 50 μM). **(B)**. Bioinformatics prediction of a putative HIF-1*α* binding motif (5’-AGGTGC-3’) within the PD-L1 promoter region using JASPAR (MA01106.2). **(C)**. Schematic representation of the PD-L1 promoter, the predicted HRE (5’-AGGTGC-3’) is located at −337/−332 bp relative to the TSS. The ChIP-qPCR amplicon spanning the HRE region is indicated. **(D)**. ChIP-qPCR analysis of HIF-1*α* binding to the PD-L1 promoter region. **(E)**. Dual-luciferase reporter assay in SK-N-SH cells transfected with wild-type (WT), HRE-mutant (Mut), or empty pGL3-Basic reporter constructs under normoxia and hypoxia. *p < 0.05, **p < 0.01, ***p < 0.001.

### 3.3 HIF-1*α* directly activates the PD-L1 promoter via the identified HRE

JASPAR analysis (MA01106.2) predicted a HIF-1*α* binding motif (5’-AGGTGC-3’) at −337 to −332 bp upstream of the PD-L1 TSS (Figures 3B,C). ChIP-qPCR showed HIF-1*α* enrichment at this region, which increased ∼3-fold under hypoxia compared with normoxia; no enrichment was detected at the distal control region (Figure 3D). In dual-luciferase assays, hypoxia strongly induced the WT promoter (3.98 ± 0.20 vs. 1.00 ± 0.05, *p* < 0.001), whereas this induction was abolished in the HRE mutant (1.16 ± 0.06 vs. 1.02 ± 0.06, ns). Empty pGL3-Basic showed negligible activity under both conditions (Figure 3E). Together, these data demonstrate that HIF-1*α* directly binds to and activates the PD-L1 promoter through this HRE site.

### 3.4 NB cells pretreated with hypoxia induce CD8^+^ T cell dysfunction/exhaustion-like phenotype via the HIF-1*α*/PD-L1 axis

SK-N-SH cells subjected to different pretreatments were co-cultured with activated CD8+ T cells for 48 h, and CD8^+^ T cell dysfunction/exhaustion-like phenotypes were analyzed by flow cytometry. Compared with the normoxic group, the proportion of PD-1^+^TIM-3^+^ double-positive T cells in the hypoxia-pretreated group increased from 5.49% ± 1.00% to 32.24% ± 3.80%. The siControl and Isotype groups showed no significant differences compared to the hypoxia model group. Following HIF-1*α* knockdown, the proportion of PD-1^+^TIM-3^+^ cells decreased to 13.10% ± 1.80%, and after PD-L1 blockade, it decreased to 11.94% ± 1.50%. Analysis of the relative mean fluorescence intensity (rMFI) for each dysfunction marker yielded consistent results: in the hypoxia model group, the rMFI for PD-1 and TIM-3 increased to 4.25 ± 0.48 and 3.68 ± 0.42, respectively, whereas after HIF-1*α* knockdown or PD-L1 blockade, both values significantly decreased to levels close to those under normoxic conditions (Figures 4A–C).

**Figure 4:**
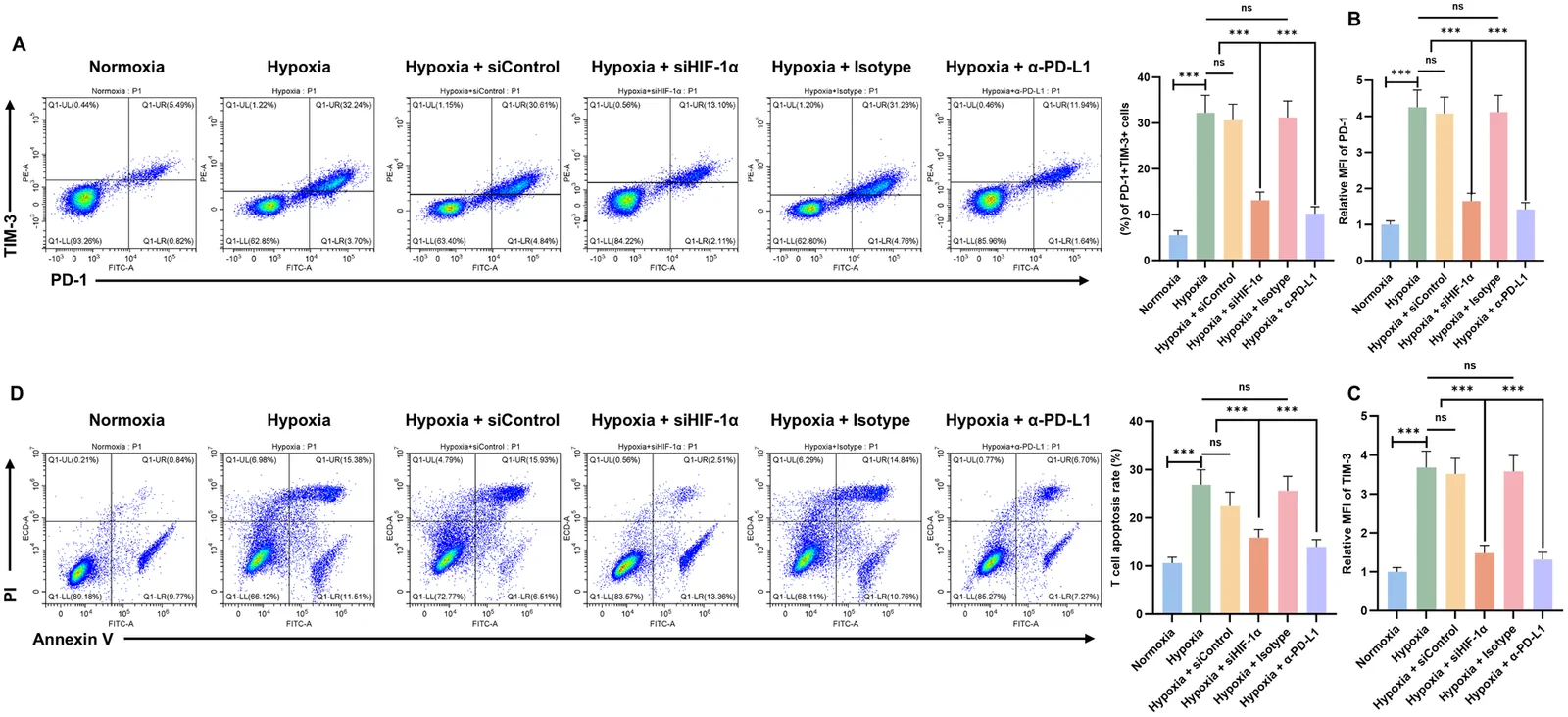
Hypoxia-pretreated SK-N-SH cells induce CD8^+^ T cell dysfunction/exhaustion-like phenotype via the HIF-1*α*/PD-L1 axis. **(A)**. Percentage of PD-1^+^TIM-3^+^ double-positive T cells. **(B**-**C)**. Relative mean fluorescence intensity (rMFI) of PD-1 and TIM-3. **(D)**. T cell apoptosis rate. PD-1/TIM-3 expression was analyzed on live CD8^+^ T cells. ***p < 0.001.

Apoptosis assays revealed that following co-culture with hypoxia-pretreated NB cells, the apoptosis rate of CD8+ T cells increased from 10.61% ± 1.20% to 26.89% ± 3.10%; after HIF-1*α* knockdown or PD-L1 blockade, it decreased to 15.87% ± 1.70% and 13.97% ± 1.50%, respectively (Figure 4D).

### 3.5 NB cells pretreated with hypoxia impair the antitumor function of CD8^+^ T cells via the HIF-1*α*/PD-L1 axis

T-cell function was assessed using a CFSE/PI cytotoxicity assay. Compared with the normoxic pretreatment group, the specific cytotoxicity rate in the hypoxic pretreatment group decreased from 79.94% ± 4.20% to 38.40% ± 2.50%. This rate recovered to 70.81% ± 3.60% after HIF-1*α* knockdown and to 76.60% ± 3.80% after PD-L1 blockade, showing no statistically significant difference compared to the normoxic group (Figures 5A,B).

**Figure 5:**
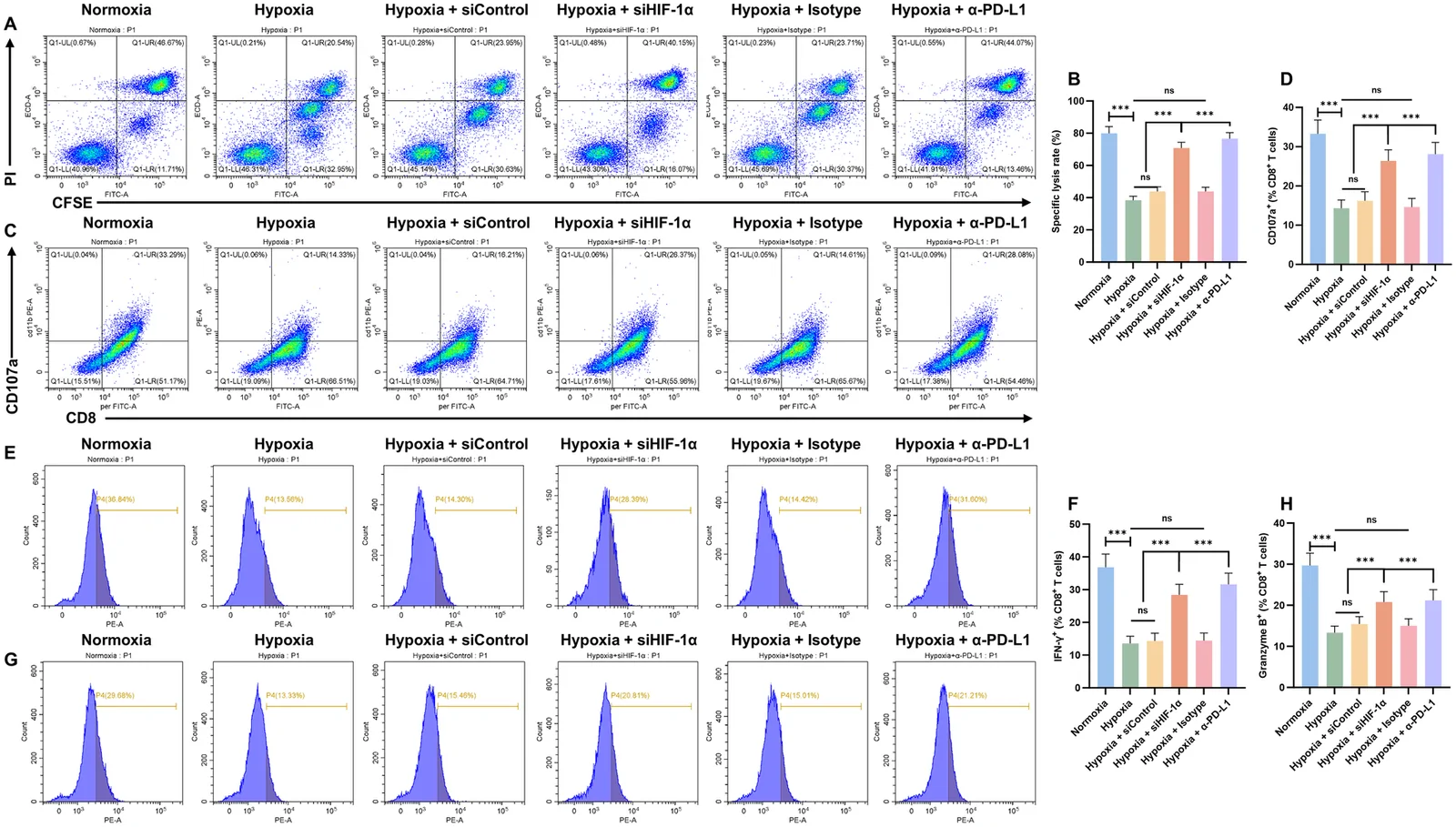
SK-N-SH cells pretreated with hypoxia suppress the antitumor function of CD8+ T cells via the HIF-1*α*/PD-L1 axis. **(A**-**B)**. Specific killing rate. **(C**-**D)**. Percentage of CD107a-positive cells. **(E**-**F)**. Percentage of IFN-*γ*-positive cells. **(G**-**H)**. Percentage of Granzyme B-positive cells. ***p < 0.001.

CD107a degranulation analysis showed that the proportion of CD107a^+^CD8^+^ T cells in the hypoxia-pretreated group decreased from 33.29% ± 3.50% to 14.33% ± 2.10%, and recovered to 26.37% ± 2.80% after HIF-1*α* knockdown, and to 28.08% ± 3.00% after PD-L1 blockade (Figures 5C,D).

Intracellular cytokine staining revealed that the proportion of IFN-*γ*^+^CD8^+^ T cells in the hypoxia-pretreated group decreased from 36.84% ± 4.00% to 13.56% ± 2.20%, while it increased to 28.39% ± 3.20% after HIF-1*α* knockdown and to 31.6% ± 3.40% after PD-L1 blockade (Figures 5E,F). The proportion of Granzyme B^+^CD8^+^ T cells decreased from 29.68% ± 3.00% to 13.33% ± 1.60%, increased to 20.81% ± 2.50% after HIF-1*α* knockdown, and to 21.21% ± 2.60% after PD-L1 blockade (Figures 5G,H). The siControl and Isotype groups showed no significant differences from the hypoxia model group in any functional parameters.

### 3.6 Targeting HIF-1*α* or blocking PD-L1 alleviates hypoxia-pretreatment-induced CD8^+^ T cell dysfunction/exhaustion-like phenotype and functional suppression

In the rescue experiment, SK-N-SH cells were treated with hypoxia for 48 h in the presence of either YC-1 (25 μM) or a PD-L1 neutralizing antibody (10 μg/mL), and then co-cultured with activated CD8^+^ T cells. Western blot results showed that YC-1 treatment reduced HIF-1*α* and PD-L1 protein expression from 13.03 ± 0.55-fold and 6.10 ± 0.32-fold to 4.91 ± 0.25-fold and 3.86 ± 0.20-fold, respectively (Figure 6A). Following co-culture, flow cytometry analysis revealed that the proportion of PD-1^+^TIM-3^+^ cells was 33.20% ± 3.80% in the hypoxia model group, 10.45% ± 2.00% in the YC-1 rescue group, and 9.58% ± 1.50% in the *α*-PD-L1 rescue group (Figure 6B). The specific killing rate recovered from 44.36% ± 2.50% to 70.41% ± 3.50% (YC-1) and 72.04% ± 3.80% (*α*-PD-L1) (Figure 6C). These results suggest that, in this co-culture model, pharmacological inhibition of HIF-1*α* or blockade of PD-L1 attenuate hypoxia-pretreatment-induced CD8^+^ T cell dysfunction/exhaustion-like phenotype and partially restores T cell function.

**Figure 6:**
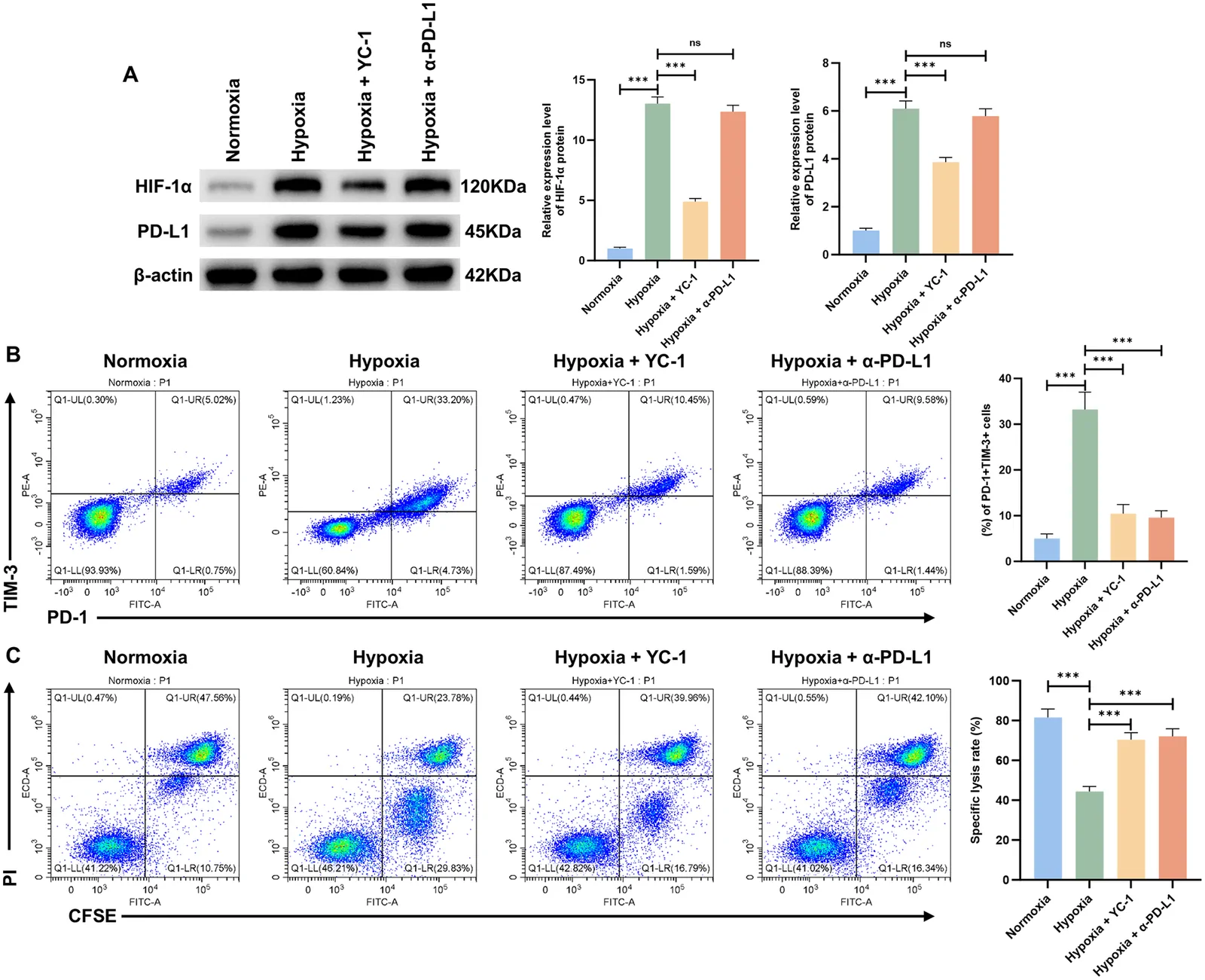
Targeting HIF-1*α* or blocking PD-L1 attenuates hypoxia-pretreatment-induced CD8^+^ T cell dysfunction and functional suppression. **(A)**. Western blot analysis of HIF-1*α* and PD-L1 protein expression levels in the normoxic group, the hypoxia group, and the hypoxia + YC-1 rescue group. **(B)**. Flow cytometry analysis of the percentage of PD-1^+^TIM-3^+^ double-positive cells among CD8^+^ T cells in each group after co-culture. **(C)**. CFSE/PI assay to determine the specific killing rate of tumor cells by CD8^+^ T cells in each group. ***p < 0.001.

### 3.7 Direct effect of YC-1 on CD8^+^ T cells

To assess whether YC-1 directly affects T cells, primary human CD8^+^ T cells were treated with YC-1 (0, 10, 25, 50 μM) for 48 h. At 25 μM, the concentration used for tumor cell pretreatment, YC-1 did not significantly affect T cell viability or IFN-*γ* production, whereas 50 μM reduced both (Supplementary Figures S3A,B). These data suggest that, at the concentration used in this study, YC-1 does not exert a major direct effect on CD8^+^ T cells, and the observed restoration of T cell function is likely mediated mainly through inhibition of HIF-1*α* in tumor cells.

## 4 Discussion

This study reveals how hypoxic conditioning of NB cells drives immune suppression through the HIF-1*α*/PD-L1 axis. We showed that hypoxia stabilizes HIF-1*α*, which directly binds to the predicted HRE motif within the PD-L1 promoter, as verified by ChIP-qPCR and HRE-mutant dual-luciferase reporter assays, and transcriptionally activates PD-L1. In co-culture experiments, hypoxia-pretreated NB cells induced a CD8^+^ T cell dysfunction/exhaustion-like phenotype and impaired antitumor function. HIF-1*α* inhibition or PD-L1 blockade attenuated these effects and partially restored T cell function. These findings provide cellular immunology evidence linking hypoxic conditioning of tumor cells to immune suppression in NB and support targeting the HIF-1*α*/PD-L1 axis.

Hypoxia is common in solid tumor microenvironments (21). Because tumor cells grow fast and blood vessels develop abnormally, NB tissues often have clear hypoxic areas (22). Inside those areas, HIF-1*α* becomes stable due to reduced prolyl hydroxylase activity and moves into the nucleus (23). In our study, HIF-1*α* protein peaked at 24 h of hypoxia, and PD-L1 levels increased gradually with longer hypoxia exposure. We further validated PD-L1 upregulation in two additional NB cell lines with different MYCN status, SK-N-BE(2) and SK-N-AS, suggesting that this response is not restricted to SK-N-SH cells. Knocking down HIF-1*α* or treating with YC-1 stopped the PD-L1 rise. Our results extend the earlier work by Noman and colleagues in mouse MDSC cells (17) to human NB cells. They also agree with recent reports in head and neck squamous cell carcinoma (24), colorectal cancer (25), breast cancer (26), and nasopharyngeal carcinoma (27). Together, these studies indicate that the HIF-1*α*/PD-L1 axis is a conserved pathway driving hypoxia-related immune escape. We also observed that PD-L1 levels gradually declined after reoxygenation, indicating that hypoxia-induced PD-L1 upregulation is reversible.

This study further demonstrated through functional experiments that hypoxia-pretreated NB cells directly induce CD8^+^ T cell dysfunction/exhaustion-like phenotype by upregulating PD-L1. Following co-culture with hypoxia-pretreated NB cells, T cells exhibited a typical dysfunctional phenotype: the proportion of PD-1^+^TIM-3^+^ cells increased approximately 5.9-fold (from 5.49% to 32.24%), the apoptosis rate increased approximately 2.5-fold (from 10.61% to 26.89%), the IFN-*γ*^+^ and Granzyme B^+^ positivity rates decreased by approximately 63% and 55%, respectively, and the specific killing rate decreased by approximately 52%. PD-1/TIM-3 expression was analyzed within the live CD8^+^ T cell gate after excluding dead cells, whereas apoptosis was measured separately; these two phenotypes are not necessarily overlapping. However, after knocking down HIF-1*α* in NB cells or using PD-L1 neutralizing antibodies, all of the above indicators were significantly attenuated, though they did not fully recover to normoxic levels. These results suggest that T-cell suppression observed in this co-culture system is driven by the HIF-1*α*/PD-L1 axis within hypoxia-preconditioned NB cells. Of note, this experimental setup does not allow us to assess the direct biological effects of hypoxia on CD8^+^ T cells, as T cells were cultured under normoxic conditions throughout the assay. In addition, we confirmed that YC-1 at the concentration used (25 μM) did not significantly affect CD8^+^ T-cell viability or IFN-*γ* production, suggesting that the restoration of T-cell function is mainly mediated through inhibition of HIF-1*α* in tumor cells. It is worth noting that considering the short-term 48-h co-culture setting, these alterations represent a CD8^+^ T-cell dysfunction/exhaustion-like phenotype, rather than bona fide chronic T-cell exhaustion driven by sustained antigen stimulation. Upregulation of PD-1 and TIM-3 may also occur during acute T-cell activation or cellular stress; definitive evidence for bona fide exhaustion requires prolonged antigenic stimulation, transcriptomic profiling, and assessment of additional inhibitory receptors (19).

In the NB microenvironment, T cell dysfunction is driven by multiple factors. Single-cell transcriptomics revealed that T cells in NB are dysfunctional and identified the NECTIN2-TIGIT axis as a key immune checkpoint. Combining TIGIT and PD-L1 blockade induced complete tumor regression in animal models (28). Tumor hypoxia can also activate the integrated stress response transcription factor ATF4, causing mitochondrial oxidative stress and death of CD8^+^ tumor-infiltrating lymphocytes, which drives terminal dysfunction (29). Moreover, a pan-cancer analysis showed that a STMN2-expressing tumor-associated macrophage subset closely interacts with dysfunctional CD8^+^ T cells; silencing BCAP31 in macrophages pushed CD8^+^ T cells toward an effector phenotype (30). Another single-cell study of NB bone marrow metastases also found enrichment of dysfunctional T cells and increased Tregs (31). These studies together paint a complex picture of T cell dysfunction regulation in NB. Our work focuses on NB cell-derived PD-L1 and demonstrates that PD-L1 upregulation on tumor cells themselves is a key step in hypoxia-preconditioned T cell inhibition, complementing other hypoxia-related immunosuppressive mechanisms such as affecting T cell metabolism or inducing M2-type macrophages (32).

Our rescue experiment provides *in vitro* proof of concept for clinical translation. Both YC-1 (HIF-1*α* inhibitor) and anti-PD-L1 antibody significantly attenuated T cell dysfunction and partially restored killing function. At the concentration used, YC-1 did not exert a major direct effect on CD8^+^ T cells, supporting that its effect is mainly through tumor cell HIF-1*α* inhibition. Blocking PD-L1 worked slightly better than HIF-1*α* inhibition. Still, even after PD-L1 blockade, the killing rate remained significantly lower than that of the normoxic control, suggesting that PD-L1 is just one node in the hypoxia-driven immunosuppressive network. Clinically, the objective response rate of anti-PD-1/PD-L1 monotherapy in high-risk NB is far lower than in adult cancers, partly because NB is an immunologically “cold” tumor (33). Recent strategies targeting hypoxia/HIF-1*α* have shown promise in NB. For example, the BET inhibitor JQ1 suppressed hypoxia-induced HIF-1*α* and enhanced anti-PD-1 efficacy in NB mouse models (20). Dual blockade of TIGIT and PD-L1 also worked well in NB (28). Therefore, combining HIF-1*α* inhibition with PD-L1 blockade (or additional TIGIT blockade) may yield additive or synergistic therapeutic benefits in pre-clinical NB models, offering a potential strategy to alleviate tumor-cell-derived immune suppression.

Our study has several limitations. First, all experiments were done *in vitro* using co-culture systems; we lack *in vivo* animal model validation. Future studies should test the combined therapy in NB xenograft or transgenic mouse models. Second, functional T-cell co-culture assays were exclusively performed in SK-N-SH cells. Although hypoxia-driven PD-L1 upregulation was confirmed in SK-N-BE(2) (MYCN-amplified) and SK-N-AS (MYCN-non-amplified) NB cell lines, further functional validation across additional NB models would strengthen our conclusions. Third, tumor cells were pre-conditioned under hypoxia and then co-cultured with T cells under normoxia; therefore, this model does not fully recapitulate a true hypoxic tumor microenvironment, and the direct effect of hypoxia on T cells was not evaluated. Future studies using true hypoxic co-culture systems are warranted. Of note, this 48-h co-culture system is insufficient to model bona fide chronic T-cell exhaustion, and we could only detect an exhaustion-like dysfunction phenotype in CD8^+^ T cells. Finally, the primary human CD8^+^ T cells used in this study were commercially sourced primary human CD8^+^ T cells; the exact number of individual donors is not disclosed by the vendor. This precludes evaluation of inter-donor variability, which represents a limitation for interpreting T-cell-related readouts. In addition, systemic toxicity of HIF-1*α* inhibitors is a concern for clinical translation, and tumor-specific delivery systems or safer inhibitors should be explored in future studies.

## 5 Conclusion

This study demonstrates that hypoxic conditioning of neuroblastoma cells stabilizes HIF-1*α*, which directly binds to the HRE motif of the PD-L1 promoter to elevate PD-L1 expression. Upregulated PD-L1 on NB cells contributes to a CD8^+^ T-cell dysfunction/exhaustion-like phenotype and impaired T-cell-mediated antitumor function in this *in-vitro* co-culture model. Either genetic knockdown or pharmacological inhibition of HIF-1*α*, as well as PD-L1 blockade, can attenuate such immune-suppressive phenotypes and partially restore T-cell function. Collectively, our findings provide *in vitro* evidence that tumor-cell hypoxic conditioning contributes to immune suppression via the HIF-1*α*/PD-L1 axis in neuroblastoma.

## Data Availability

The raw data supporting the conclusions of this article will be made available by the authors, without undue reservation.
