# Supplementary material for: Hypoxic conditioning of neuroblastoma cells promotes CD8+ T-cell dysfunction through the HIF-1α/PD-L1 axis
Source: Front Pediatr. 2026 Oct 14;14:1923893. doi: 10.3389/fped.2026.1923893 (PMC13713678; doi:10.3389/fped.2026.1923893)
Supplement: Supplementary file 1 [file Datasheet1.zip › Supplementary materials.doc]

**Figure S1. Hypoxia induces PD-L1 upregulation in additional neuroblastoma cell lines.** A. Relative PD-L1 mRNA expression levels in SK-N-BE(2) (MYCN-amplified) and SK-N-AS (MYCN-nonamplified) cells. B. Relative PD-L1 protein expression levels in SK-N-BE (2) (MYCN-amplified) and SK-N-AS (MYCN-nonamplified) cells. ^**^*p* < 0.01, ^***^*p* < 0.001.

**Figure S2. Time course of PD-L1 expression in SK-N-SH cells under normoxic conditions and its recovery following hypoxia.** A. PD-L1 mRNA expression levels in SK-N-SH cells cultured under normoxic conditions for 0, 6, 12, 24, and 48 hours. B. PD-L1 protein expression levels in SK-N-SH cells cultured under normoxic conditions for 0, 6, 12, 24, and 48 hours. C. PD-L1 mRNA expression levels in SK-N-SH cells 0, 6, 12, and 24 hours after reoxygenation following 24 hours of hypoxia. D. PD-L1 protein expression levels in SK-N-SH cells 0, 6, 12, and 24 hours after reoxygenation following 24 hours of hypoxia. ^*^*p* < 0.05, ^**^*p* < 0.01.

**Figure S3. Direct effect of YC-1 on CD8⁺ T cell viability and IFN-γ production.** Primary human CD8⁺ T cells were treated with YC-1 (0, 10, 25, 50 μM) for 48 h. A. Cell viability measured by CCK-8 assay. B. IFN-γ levels in culture supernatants measured by ELISA after PMA/ionomycin stimulation for 4-6 h. ^*^*p* < 0.05.
